# Integration of Genome Scale Metabolic Networks and Gene Regulation of Metabolic Enzymes With Physiologically Based Pharmacokinetics

**DOI:** 10.1002/psp4.12230

**Published:** 2017-09-08

**Authors:** Elaina M. Maldonado, Vytautas Leoncikas, Ciarán P. Fisher, J. Bernadette Moore, Nick J. Plant, Andrzej M. Kierzek

**Affiliations:** ^1^ School of Biosciences and Medicine Faculty of Health and Medical Sciences, University of Surrey Guildford Surrey UK; ^2^ Quantitative Systems Pharmacology Simcyp Limited (A Certara Company), Blades Enterprise Centre Sheffield UK; ^3^ Translational Science and DMPK Simcyp Limited (A Certara Company), Blades Enterprise Centre Sheffield UK; ^4^ School of Food Science and Nutrition Faculty of Mathematics and Physical Sciences, University of Leeds Leeds UK; ^5^ School of Molecular and Cellular Biology, Faculty of Biological Sciences, University of Leeds Leeds UK

## Abstract

The scope of physiologically based pharmacokinetic (PBPK) modeling can be expanded by assimilation of the mechanistic models of intracellular processes from systems biology field. The genome scale metabolic networks (GSMNs) represent a whole set of metabolic enzymes expressed in human tissues. Dynamic models of the gene regulation of key drug metabolism enzymes are available. Here, we introduce GSMNs and review ongoing work on integration of PBPK, GSMNs, and metabolic gene regulation. We demonstrate example models.

Physiologically based pharmacokinetic (PBPK) modeling has delivered considerable impact in drug development and has become the industry standard for prediction of drug‐drug interactions (DDIs), formulation effects, and pharmacokinetics in human populations. As a bottom‐up, literature‐based, mechanistic computer simulation approach, it shares general methodology with the computational systems biology, in which molecular biology knowledge is assimilated into molecular network models. In particular, reconstruction of genome scale metabolic networks (GSMNs) has led to mechanistic models incorporating a whole set of metabolic enzymes expressed in human tissues. Moreover, dynamic models of the expression of key drug metabolism enzymes are available. Currently, PBPK models account for about 20 genes involved in drug metabolism, already allowing prediction of the impact of genetic variability on drug population pharmacokinetics (PKs) as well as identification of individual genetic backgrounds leading to drug concentrations outside of the therapeutic window. The scope of this pharmacogenetic modeling can be naturally expanded by assimilation of GSMNs as mechanistic models of tissue intracellular space compartments within PBPK. Genetic polymorphism in thousands of metabolic enzyme genes accounted for by GSMN can be mechanistically linked to drug PKs, bringing PBPK to the pharmacogenomic domain. Mechanistic models of the intracellular space can be further expanded by assimilation of the dynamic models of gene regulation, which increasingly becoming available for key drug metabolism enzymes. Although these models add only a few genes, they account for complex dependencies of drug metabolism enzyme activities on endogenous metabolites, such as cortisol. Moreover, drug‐drug interactions (DDIs) involving compounds, such as pregnane X receptor (PXR) ligands, which bind to transcription factors rather than metabolic enzymes, can be modeled with more mechanistic detail.

In this tutorial, we will describe how the scope of PBPK models can be extended by accounting for whole‐cell metabolism and gene regulation of key drug metabolism enzymes. We assume that the reader is familiar with ordinary differential equations (ODEs) and we will introduce PBPK very briefly. The PBPK tutorial has been previously published.^1^ We will introduce GSMNs in much more detail as this is an emerging approach in the systems pharmacology field. A brief review of dynamic models available for drug metabolism enzyme genes will be also provided. We will then proceed to describe the current state of the art in integration of GSMNs and PBPK. Subsequently, we will provide a working example model in which liver‐specific GSMN is integrated with a general PBPK model and a model of the induction of drug metabolizing enzyme by cortisol and PXR ligands. The example will focus on identification of genes affecting the PK of toxic drug metabolites. We hope that this tutorial will draw the attention of the modeling community in pharmaceutical industry to new avenues of mechanistic modeling opened by molecular network models developed in systems biology.

## PHYSIOLOGICALLY BASED PHARMACOKINETICS: A BOTTOM‐UP MECHANISTIC MODELING

The wide adoption of PBPK in drug development demonstrates the value of bottom‐up, whole‐system scale, and the literature‐based mechanistic modeling approach. Current PBPK models are composed of hundreds of ODEs assimilating literature knowledge and data on the physiological processes involved in drug absorption, distribution, metabolism, and excretion. For example, simulation of full PBPK model available in the Simcyp simulator involves about 450 ODEs, which have been defined and parameterized by research and development teams scrutinizing about 17,000 publications since 2012. The PBPK models provide whole‐system scale description in a sense of including all major physiological compartments in the body. The granularity of this description is being continuously increased by versions of the model expanding mechanistic detail of selected compartments. For example, to model oral drug administration, the gut compartment may be split into lumen (unabsorbed drug) and enterocyte (absorbed drug). As the models advance, each compartment is further split into a number of subcompartments describing different regions of the gastrointestinal tract, such as the stomach, duodenum, jejunum, ileum, cecum, and colon. Likewise, other compartments, such as the liver and the lungs may be further divided. Incorporation of additional mechanistic detail into the model poses a challenge, as the values for additional parameters need to be determined either from literature or parameter estimation from available data. On the other hand, mechanistic detail enables extrapolation from *in vitro* data – some model parameters are intrinsic properties of tissues or enzymes that can be measured *in vitro* without the need of animal or clinical studies. Furthermore, increasing the granularity of the model may be essential when a particular application requires the presence of certain variables that influence the compound PKs being investigated. For example, simulation of DDI requires a resolution of individual enzymes and transporters that compounds bind to. Last but not least, mechanistic detail allows incorporation of parameter values and their distributions in populations for subsequent prediction of compound PK variability in the specific target population. For example, the model that resolves liver and kidney clearance can be used for simulation of populations with liver or kidney impairment. Correlations among body weight, age, and blood flows can be used to simulate virtual clinical trials and improve experimental design. Therefore, although the level of abstraction in a mathematical model is an arbitrary decision of its authors, an increase of granularity increases the scope of questions the model can potentially address. It is also generally accepted that mechanistic models are more likely to extrapolate and predict beyond the data used for parameter estimation than abstract phenomenological models.

The PBPK models, as the name implies, are concerned with physiological rather than molecular level variables. The notable exception is drug metabolizing enzymes and drug transporters, which are described by mechanisms of compound binding, transport, and enzymatic reaction, familiar to a biochemist. Apart of application in DDI, mentioned above, molecular level mechanistic detail enables accounting for genetic polymorphism. For a number of molecular variants, the binding constants and maximal velocities have been determined *in vitro*. Therefore, data on allele frequencies in the population can be used in virtual trial simulation. In personalized medicine context, patient's genotype can be used to inform decisions about individual dosing regimen. This in turn opens an avenue for future assimilation of the wealth of information resulting from genomics revolution, such as the 100,000 genomes project already funded by the United Kingdom government.^2^ This in turn, brings PBPK close to systems biology, in which mechanistic modeling of genotype‐phenotype relationship is a prominent interest. Mechanistic models of intracellular networks created in the systems biology field are a natural, further mechanistic extension of PBPK, as they provide detailed description of intracellular space, which has been so far modeled as a uniform, well‐stirred compartment. Assimilation of these models would immediately provide more variables directly associated with genes, thus extending the scope of pharmacogenomic predictions and extending the use of genomics data in simulation of populations and individuals.

## GENOME SCALE METABOLIC NETWORKS

The reconstruction and constrained‐based modeling of GSMNs is an area of computational systems biology, which to a remarkable extent shares methodology of the PBPK. Like PBPK models, the GSMNs are large, whole‐system scale models created by assimilation of scientific literature describing mechanistic detail. In the case of GSMNs, the system under consideration is metabolism of a living cell (i.e., the system of coupled, enzyme catalyzed, chemical reactions providing energy, building blocks, and reducing power for the cell). Although quantitative parameterization of the ODEs representing whole‐system models involving several thousands of chemical reactions is still not possible, the constraint‐based modeling (CBM)^3^, ^4^ approach allows quantitative analysis of metabolic flux distribution at steady state. This enables investigation of the effects of genetic polymorphism for a whole set of human metabolic genes as well as mechanistic interpretation of gene expression data.

### Flux balance analysis: A constraint‐based approach

The GSMN is a system of thousands of coupled chemical reactions involving thousands of metabolites. Metabolism is the best studied organization level of the cell and there is sufficient knowledge to write chemical reaction formulas defining connectivity and stoichiometry of this system. However, quantitative parameterization of the ODE model, simulating time evolution of each metabolite concentration is still not possible. The flux balance analysis (FBA)^4^ allows modeling of the steady‐state distribution of reaction fluxes, without the knowledge on kinetic parameters and initial concentrations required by an ODE model, which is sufficient for prediction of the metabolic conversions feasible in GSMN. Apart of qualitative prediction of metabolic function feasibility, quantitative prediction of reaction fluxes is also possible, if sufficient data are available for rate limiting nutrient transport uptake and secretion rates. The FBA has been first proposed by Fell & Small^5^ to study fat synthesis in adipose tissue. Early introduction to the method can be found in the classical metabolic engineering textbook of Stephanopoulos *et al*.^6^ The study by Lewis *et al*.^7^ applied FBA to modeling whole‐cell metabolism based on genome annotation of metabolic enzymes and transporters, which has led to evolution of the prominent field of systems biology, best reflected by a phylogenetic tree of constraint‐based approaches originating from FBA.

**Figure 1:**
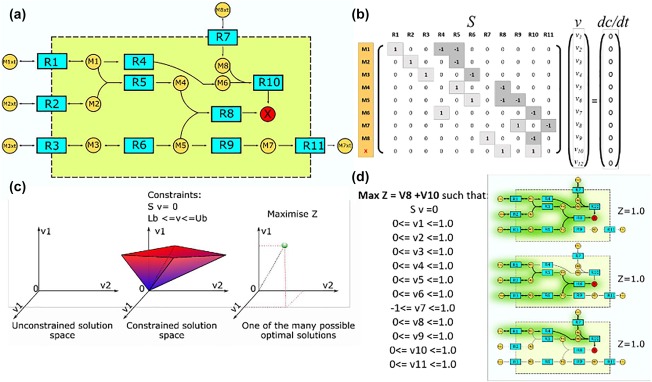
Flux balance analysis – a constraint based approach. (**a**) Example metabolic network. External metabolites representing nutrients available in cellular microenvironment are labeled by “xt.” Production of metabolite X is the metabolic capability of interest. (**b**) Stoichiometric matrix S and equation defining flux balances at steady state for network shown on **a**. Note, that external metabolites are assumed to be unbalanced sources and sinks and are not included. (**c**) In an unconstrained network, fluxes can assume any value. Imposition of steady‐state balance constraints (S v = 0) defines convex solution space. The volume of this space can be further decreased by introduction of bounds representing thermodynamic constraints on reaction reversibility as well as existing quantitative measurements of fluxes. The linear programming is used to maximize objective function Z. The value of Z is guaranteed to be maximal, but there may be many alternative solutions v with the same maximal value of Z. (**d**) Determination of the maximal synthesis rate of metabolite X in an example network shown in **a**. Reaction v_7_ is assumed to be reversible, all other reactions are irreversible. No quantitative measurement of fluxes is available, so arbitrary value of 1 is used as a flux bound. There are three alternative solutions with the same maximal value of objective function. Further constraints would decrease degeneracy of the solution. For example, measurement of M1xt, M2xt, M3xt, and M4xt consumption/release would allow constraining of transport reactions R1, R2, R3, and R7, which would lead to one of the three solutions having the largest value of Z. Reaction R1 (red box) is essential for production of X. Constraining of R1 to (0, 0) will make all three solutions not feasible. The network is robust, only R1 is essential. Any other single reaction deletion would not abolish production of X.

To introduce the mathematical formulation of FBA, let us consider the system of N coupled chemical reactions involving M metabolites (**Figure** 1). Chemical reaction formulas with stoichiometric coefficients are represented by M × N stoichiometric matrix S, where S_i,j_ is a stoichiometric coefficient of metabolite i in reaction j. The variables of an FBA model are reaction fluxes in concentration per time units, rather than metabolite concentrations. Let v be a vector of M reaction fluxes and v_i_ the flux of reaction i. At steady state, the FBA model is defined by the following equation:

(1) $$\frac{dc}{dt}=0=Sv$$

where c is a vector of M metabolite concentrations. Therefore, an FBA model is represented by a system of ODEs at steady state. When reaction fluxes, rather than concentrations, are used as variables, the linear model is obtained. This model defines the convex solution space containing all flux distributions v satisfying Eq. (1). The solution space constitutes a null space of stoichiometric matrix and a variety of linear algebra approaches have been introduced to explore its properties. This includes formal definitions of metabolic pathways based on analysis of support vectors.^8^, ^9^ In more intuitive terms, Eq. (1) states that for each metabolite the sum of fluxes producing and consuming this metabolite equals 0 – a formal statement of steady‐state assumption. The flux balance equations constrain the solution space by allowing only flux vectors v, where production and consumption fluxes balance for each metabolite. Hence, the method is referred to as an FBA and together with approaches that extend it by incorporation of other constraints is known in systems biology as CBM.

In most applications, the linear system defined by Eq. (1) is underdetermined. There are not enough reactions for which fluxes have been measured to determine the remaining flux values by inversion of matrix S. Instead, linear programming (LP) is used to determine whether specific metabolic function is feasible and what would be its maximal capacity, given model constraints. The following LP problem is solved:

(2) $$\begin{array}{c} Maximise:\;z=\;\overset{N}{\underset{i=1}{\sum }}a_{i}v_{i}\\ \text{Such}\;\text{that}:\\ 0=Sv\\ l_{i}<v_{i}\leq u_{i} \end{array}$$

where z is an objective function, a_i_ is an objective function coefficient for reaction i, l is a vector of lower bounds of N reactions, and u is a vector of upper bounds of N reactions. The LP solver finds maximal value of z given flux balance constraints defined by Eq. (1) and arbitrary, additional constraints defined as flux bounds. Because this is a linear problem with a convex solution space, the solver is guaranteed to find the maximal possible value of z. However, there may be many different solutions for v, for which z assumes its maximal value.

The objective function z represents metabolic function of interest defined as a linear combination of reaction fluxes. For example, z could be a sum of fluxes producing adenosine triphosphate, bile acid secretion flux, or biomass reaction flux. Even if there is no quantitative information about any of the fluxes, LP maximization of z would test stoichiometric feasibility of the metabolic function of interest. The existence of the feasible solution where z ≠ 0 would indicate that metabolic function can be realized. Otherwise, metabolic function is not possible in the system of stoichiometric balanced, coupled chemical equations. This qualitative simulation can be already used to make useful predictions. For example, “druggable” metabolic vulnerabilities of cancer cell lines or bacterial pathogens can be identified by *in silico* essentiality screen, in which each reaction is removed from the model and feasibility of biomass synthesis is tested.^10^ If quantitative measurements are available for some of the fluxes, those can be incorporated by setting them as flux bounds. As quantitative information is incorporated, the volume of the solution space decreases and the model gradually increases its qualitative level of detail. The ability to draw useful conclusions from qualitative data alone and then gradually introduce quantitative description, as data become available, is a major feature of FBA enabling simulation of whole‐cell scale models. The bounds are also used to set reaction reversibility, the lower bound of 0 allows only positive flux values, whereas unconstrained reaction can assume both positive and negative fluxes. Furthermore, reaction bounds can be used to implement model perturbations. In the essentiality scan example, setting reaction flux bounds to (0, 0) is equivalent to removing it from the model. Last but not least, the bounds are used to define boundary conditions of the model (i.e., sources and sinks of metabolic flux). The source is defined as the reaction that does not have balanced substrates and the sink is defined as the reaction that does not have balanced product. FBA assumes the availability of source metabolite and the capacity of the sink are unlimited. In many software and models, including our own, these reactions feature external metabolites, which make reaction formulas more readable, but do not feature in stoichiometric matrix S and do not contribute to Eq. (1). Setting the bounds of source and sink reactions determine which metabolites are available as the nutrients, which are secreted, and what is the maximal capacity of the uptake or secretion.

The LP is the most frequently used approach to explore a solution space defined by Eq. (1), but it is not the only one. Null space analysis of S has been already mentioned above,^8^, ^9^ but its disadvantages are that it does not use quantitative reaction flux bounds and leads to combinatorial explosion of alternative pathways conducting particular metabolic function. Recently, methods using Markov Chain Monte Carlo sampling of solution space gain prominence.^11^, ^12^ Briefly, the stochastic process is created, in which solutions are randomly generated within reaction flux bounds. Solutions are retained if they fulfill stoichiometric constraints defined by Eq. (1) or rejected otherwise. The sample is then analyzed to identify metabolic functions of the model. The advantage of this method over FBA is that it does not involve maximization of the objective function, which is a frequently questioned assumption, and identifies correlations across different feasible solutions. The disadvantage is the increase in computational cost and a lack of a definitive answer, which can be used as a proof. If the FBA objective function is 0, this proves that there is no solution representing a particular metabolic capability. A Monte Carlo sample does not provide such a proof and very large samples are needed to reject existence of metabolic function based on low probability.

### Reconstruction of genome scale metabolic networks

**Figure 2:**
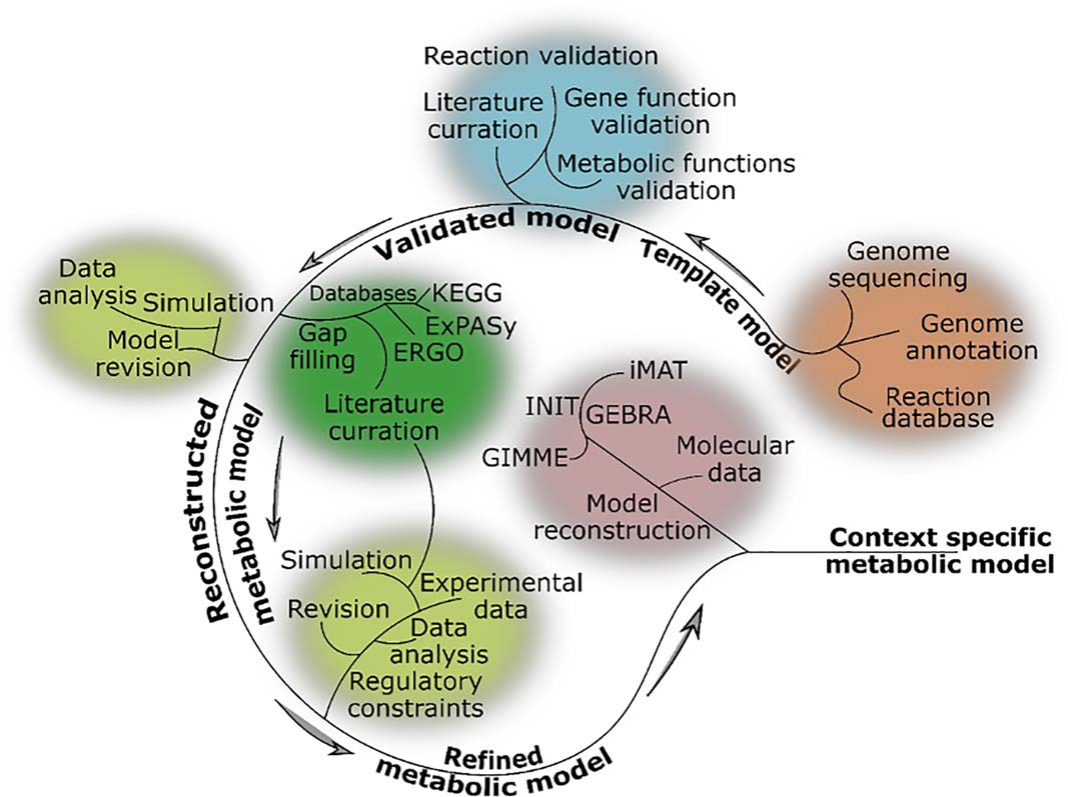
Reconstruction of genome scale metabolic networks. The process starts with full genome sequence and its annotation by sequence analysis approaches. A database of elementally and charge balance reactions, representing known activities of metabolic enzymes is required. The first step is to extract from reaction database those reactions, which are associated with enzymes encoded by the genome. Gene‐reaction association rules are created. The scientific literature is curated to identify metabolic capabilities and possibly essential genes of cell/tissue under investigation. External metabolites and transport reactions are defined and flux balance analysis simulation is used to compare predicted metabolic capabilities of the model with literature knowledge. This usually leads to the conclusion that the model requires gap filling (i.e., incorporation of enzymatic reactions for which genes are not yet identified in the organism of interest). This model revision is supported by further literature curation and exploration of metabolic reaction databases, such as KEGG or Reactome. The model reproducing basic metabolic function is then compared with further data available from literature or experiments performed within the project. Additional constraints, such as gene expression in particular tissue may be incorporated. Finally, a variety of approaches can be used to integrate transcriptomic and proteomic data and create model specific to tissue and condition (context) of interest.

The GSMN is created in the reconstruction process (**Figure** 2), in which the first step is identification of all genes in the genome of the organism of interest, which encode metabolic enzymes and transporters. This is based on existing genome annotation or additional sequence analysis and literature‐based annotation performed within reconstruction project. In the case of human and other multicellular organisms, further literature information as well as transcriptomic and proteomic data are used to determine which of the genes are expressed in the tissue of interest. Subsequently, the knowledge about metabolic and transport reactions catalyzed by products of metabolic genes is used to create the system of coupled chemical reactions representing the first draft of the GSMN. These reactions should be a stoichiometric mass and charge balanced, which requires assumption of cellular pH and additional assumptions for intracellular metabolites that involve pools of heterogeneous molecules (i.e., lipids of different hydrocarbon chain lengths). Furthermore, each reaction is set to be reversible under physiological conditions or not reversible, based on literature data or free energy calculations. Once the first draft is available, the stoichiometric matrix and reaction flux bounds are created and the FBA is used to test whether the model is capable of reproducing known metabolic conversions performed by cell/tissue under investigation. As described in the previous section, these metabolic capabilities are mathematically represented as objective functions. For example, a liver GSMN should be capable of converting lactate to glucose and producing bile acids. Frequently, the biomass reaction is formulated, which groups all metabolites that are considered to be biomass components. The flux of this reaction calculated in FBA simulation equals the growth rate of cells under investigation and can be directly compared with experimental data. This is particularly useful in modeling bacterial pathogens and cancer cell lines.

The first draft of the model based on genome annotation alone usually fails to reproduce known metabolic functions and does not represent feasible cellular metabolism. This prompts an iterative process of model refinement. The most common refinement step is gap filling. Due to errors in genome annotation, some of the reactions are missing and metabolic conversions reported in literature cannot be reproduced. The gaps are filled by introducing additional reactions, which are not associated with enzymes. These should be supported as much as possible by literature evidence on the particular metabolic step, but arbitrary assumptions are inevitable. The list of GSMN reactions for which enzymes and genes are not known in itself represents a useful set of experimental leads. Further experimental studies can be conducted to identify the enzymes and transporters catalyzing hypothetical metabolic steps.

All reactions in the GSMN, for which enzymes or transporters are known, are linked to genes in the genome by Boolean statements using gene names as variables. If the reaction is catalyzed by a multisubunit protein, the genes encoding individual subunits enter Boolean expression as terms of AND operator. If the reaction can be catalyzed by alternative enzymes, the genes are connected by OR operator. More complex Boolean expressions can be used to describe the reactions that can be catalyzed by many different multisubunit enzymes or transporters. Some modeling groups prefer to create multiple copies of reactions that can be catalyzed by multiple enzymes and use only AND statement. Some models use more complicated rules, in which genes are associated with proteins and proteins are associated with reactions. In general, we will refer to the Boolean rules describing the association between genes and reactions as gene‐protein‐reaction association rules.

## DYNAMIC MODELS OF GENE REGULATION

A major component of the body's strategy to survive in a complex chemical milieu is the use of gene regulation to allow metabolic adaptation. The body has a base metabolic state that corresponds to healthy physiology. Exposure to external chemicals, or changes in levels of endogenous chemicals, will lead to deviations from this base state, increasing the risk of adverse health outcomes. The body's response to this chemical challenge is to alter the metabolic state such that the body moves back to the base state, usually referred to as homeostasis.^13^, ^14^ This response can be divided into three stages: first, the chemical environment must be sampled; second, gene regulation must be initiated if a move away from homeostasis is detected; and third, once the environment returns to the base state, gene regulation must also return to that observed prior to the challenge.^13^, ^14^

Sampling of the chemical environment within a cell is achieved through a number of protein sensors. A major class of these sensor proteins is the ligand‐activated transcription factors, which only activate gene transcription upon binding of their specific ligands. In addition, as ligand binding is an affinity‐driven process, receptor occupancy is a determinant of the impact on the gene regulatory network (GRN). Genes encoding drug‐metabolizing enzymes and drug transporters have their expression controlled by members of the nuclear receptor family of ligand‐activated transcription factors, and these have some unique properties.^15^ To be able to sense and respond to thousands of chemicals, the nuclear receptors must show a high degree of promiscuity in their regulation of gene expression, allowing them to balance the body's response to multiple chemicals. For example, glucocorticoids may bind and activate the mineralocorticoid receptor, glucocorticoid receptor (GR), and PXR. However, affinity for these receptors varies over four orders of magnitude, with Kd values of approximately 1 nM (mineralocorticoid receptor), 10 nM (GR), and 10 μM (PXR).^16^, ^17^, ^18^ This allows different sets of GRNs to be regulated at different glucocorticoid concentrations, expanding the dynamic range over which the body can sense and respond to glucocorticoids. In addition to this property of one ligand‐many receptors, nuclear receptors also demonstrate one receptor‐many ligands; for example, the PXR may be activated by a wide range of therapeutic agents and is considered a general xenosensor.^19^ These two design properties allow nuclear receptors to sense a wide range of chemicals over a large dynamic range, and coordinate body responses to most effectively meet the chemical challenge while maintaining homeostasis.^15^ However, this promiscuity raises the potential of chemical‐drug interactions, and predicting these is an important component of the drug development process.^20^, ^21^

### Quantitative, ODE models of metabolic enzyme gene regulation

There are numerous examples of ODE‐based models that reconstruct chemical‐mediated alterations in gene expression of drug‐metabolizing enzymes. Of particular relevance to drug metabolism are interactions with the nuclear receptor PXR, which regulates the expression of many drug‐metabolizing enzymes (both phase I and II) as well as drug transporters.^15^ Bailey *et al*.^22^ examined the role of negative feedback in regulating PXR target gene expression. They demonstrated that negative feedback was an important factor to prevent hyperexpression of PXR target genes, which could cause an exaggerated response to chemical exposure. The consequence of such an exaggerated response could range from a simple increase in the time required to return to homeostasis, to premature cessation of drug action. Such negative feedback loops exist for other nuclear receptors, such as the GR, PXR, and the androgen receptor.^23^, ^24^, ^25^ In addition, there are interactions between nuclear receptors helping to coordinate the body's response to its chemical environment. For example, Kolodkin *et al*.^26^ examined the interactions between the glucocorticoid cortisol and its cognate receptors PXR and GR. They simulated both the binding of cortisol with GR (high affinity) and PXR (low affinity), as well as the impact of activated GR on the expression of GR (negative feedback^23^) and PXR (positive feedforward^27^). They demonstrated that these interactions were necessary to regulate both the amplitude and duration of the response to cortisol challenge, balancing the biological response to stress (e.g., fight or flight response) vs. the adverse health outcomes associated with prolonged cortisol stimulation (e.g., obesity, cancer, and diabetes).

The examples above demonstrate how the use of ODE‐based models of the gene regulation of drug‐metabolizing enzymes can lead to biological insights into how the body responds to chemical challenge. In addition, robustly parameterized ODE models allow the accurate reproduction of concentration‐time curves for the response to chemical challenge. This is important for predicting the body's response to both individual and concomitant exposure scenarios.

### Qualitative, nonparametric simulation of metabolic enzyme gene regulation dynamics

The reconstruction of ODE‐based networks requires a high degree of parameterization, and this may limit the scale of generated models. In addition, the time required to build such highly detailed mechanistic models is not inconsequential, and may be an important factor for individuals beginning a project. An alternate approach to highly detailed ODE models is the use of approaches that represent network connectivity in a more qualitative manner. Perhaps the simplest way to qualitatively model GRNs is through the use of the Boolean network.^28^, ^29^ Here, each gene is represented as either “active” or “inactive,” and interacts with other genes through experimentally determined molecular interactions. As coverage of the genes within an organism increases, so does the degree of interconnection within the network. The result is a complex connectivity map that can determine the state of a gene (active or inactive), dependent upon the state of its interacting genes. However, Boolean networks have a number of caveats; most notably, the exponential growth of connections as the network grows makes the model solution complex. In addition, it is difficult to represent unknown data within simple Boolean networks, meaning that problems rapidly become unfeasible for all but the best described biological networks. These problems can be addressed through the use of more complex approaches, such as probabilistic Boolean networks,^30^ or through model reduction to identify the minimal model that can reproduce the desired behavior.^31^ Such approaches have allowed the examination of the design principles behind GRNs, both in normal biology^32^, ^33^ and following drug exposure.^34^, ^35^ For example, Arshad *et al*.^34^ used a Boolean network to describe the interaction of cancer chemotherapeutics with pathways commonly mutated in breast cancer. Using this approach, they were able to predict the efficacy of combination therapies.^34^

An alternative, or at least complimentary, approach to the use of Boolean GRNs is bipartite graph approaches, such as Petri nets. They provide both an established graphical notation and formal mathematical semantics that are ideal for describing GRNs. In a Petri net, places represent species (i.e., genes, proteins, and chemicals, etc), whereas transitions represent reactions (e.g., enzymatic conversions, and translocations, etc). Places are connected to transitions via arcs, which indicate the nature of the interaction; one transition may have one or more inputs (pre‐places) and products (post‐places). Places contain tokens, with the number and location of these tokens across the network indicating its current state (or marking).^36^ Extended Petri nets add reading and inhibition arcs, representing interactions that do not consume a token upon transition firing (e.g., enzyme catalysis). Petri nets have been used to model the behavior of molecular networks, including GRNs.^37^, ^38^, ^39^, ^40^ Their application toward examining body responses to drug exposure has been more limited, although (extended) Petri nets have been used to examine more complex drug interactions, including the prediction of combination therapies.^41^ The quasi‐steady‐state Petri Net approach of Fisher *et al*.^42^ uses Petri nets to represent GRNs, which then interact with a GSMN to examine cell‐scale metabolic adaptations to a chemical challenge.^42^, ^43^ They demonstrated how interactions between nuclear receptor‐mediated GRNs and post‐translational modifications underpinned the coordination of the liver responses to cholesterol challenge.^42^

## MULTISCALE MODELS INTEGRATING PBPK, GSMN, AND GENE REGULATION OF METABOLIC ENZYMES

### Quasi‐steady‐state approximation and model‐coupling approach

**Figure 3:**
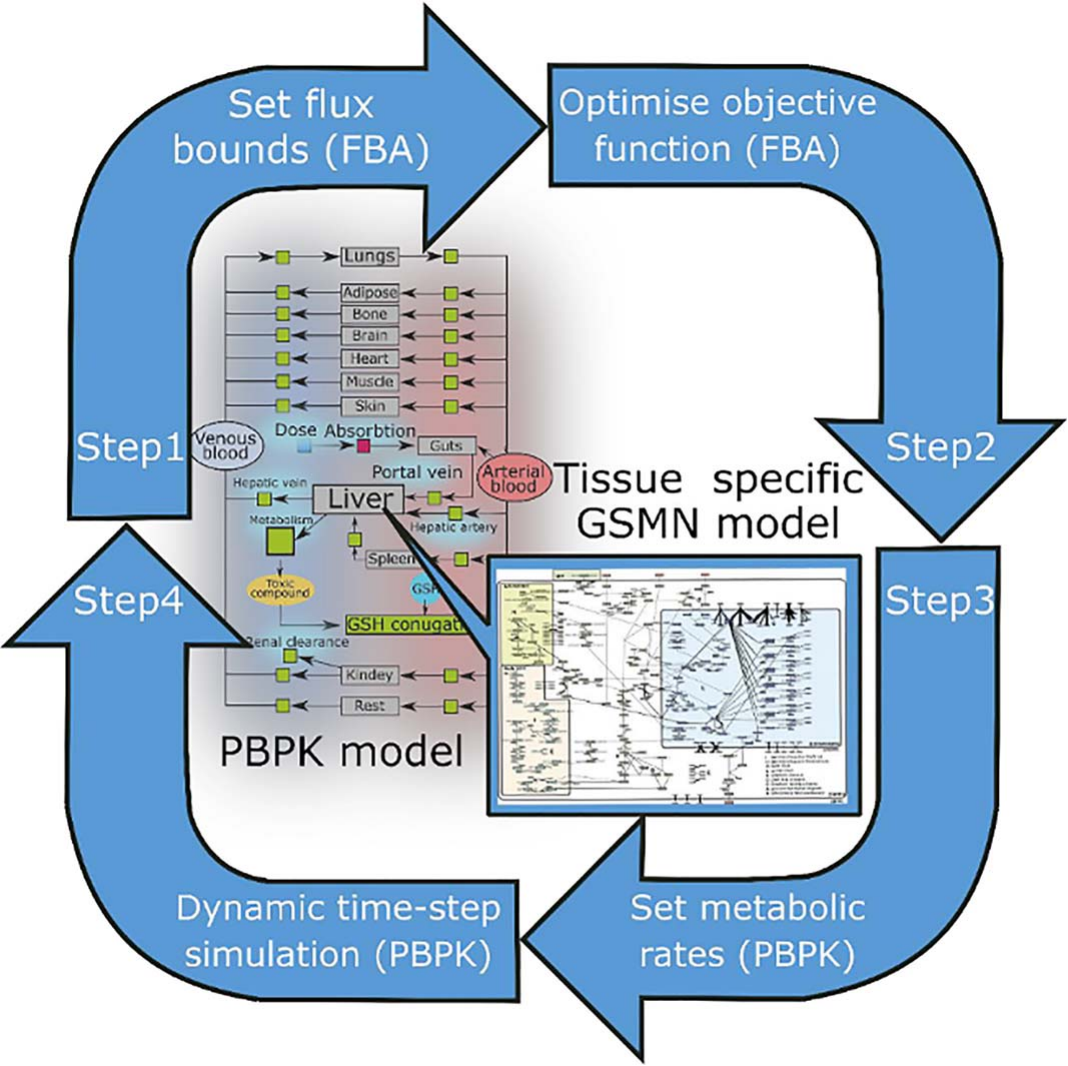
Integration of physiologically based pharmacokinetic (PBPK) and genome scale metabolic network (GSMN) in quasi‐steady‐state framework. The tissue‐specific GSMN is used as a mechanistic model of metabolism in the intracellular space of the PBPK compartment. For example, the HepatoNet1 model is used to represent liver metabolism. Simulation is performed in time‐steps, which are short compared to the timescales of drug distribution and clearance in PBPK model. The intracellular metabolism is assumed to be at quasi‐steady‐state within each time‐step. In a general case, each iteration of dynamic simulation consists of four steps. Step 1: Selected flux bounds in the GSMN model are set according to the current drug concentration and/or metabolism rate in the PBPK model. Step 2: The GSMN objective function and an example solution are calculated by flux balance analysis (FBA). Step 3: Objective function value and/or example solution fluxes are used to set metabolism rates in PBPK model. Step 4: Drug concentration and metabolism rates are updated by one time‐step of PBPK simulation. The objective functions, fluxes, and formulas used to calculate FBA bounds vary depending on specific applications. In some cases, some of the four basic steps are not executed. For example, examination of the enzyme inhibitor pharmacokinetic effect on metabolic capabilities requires only feedforward of inhibitor concentration to GSMN bounds, but does not require feedback of metabolism rates to PBPK. In this case, step 3 is not executed and the FBA objective function and fluxes are used only as simulation outputs. The case in which the GSMN is used solely to calculate the scaling factor for PBPK metabolism rate is also possible (step 1 not executed). GSH, glutathione.

In recent years, several successful multiscale modeling approaches connecting PBPK with GSMNs have been published (**Table** 1).^43^, ^44^, ^45^, ^46^ The computational approach to coupling both types of models is based on quasi‐steady‐state assumption first introduced in dynamic FBA simulation of bacterial batch cultures.^47^ Metabolic reactions in the cell are much faster than processes of drug absorption, distribution between physiological compartments, and clearance. Therefore, one can assume that a GSMN model reaches steady state within the timescale of PBPK simulation time‐step. This dictates basic model coupling strategy shown in **Figure** 3, first formulated by Krauss *et al*.^44^ First, the variables linked between two models are identified as well as equations describing their dependencies. For example, metabolic enzyme inhibitor concentration in tissue compartment calculated by a PBPK model can be linked to reaction flux bounds in GSMN through classical enzyme inhibition equation. The uptake flux calculated by a GSMN model to be required for a particular objective may be used as a drug metabolism rate in PBPK simulation. Following the terminology of Krauss *et al*.,^44^ the feedforward coupling introduces dependence of GSMN flux bound on the concentration calculated by PBPK and feedback coupling uses FBA solution to set the rates of metabolic processes in a PBPK model. During the simulation, a time‐step, typically of the order of few minutes, is introduced. Within each time‐step, four basic steps, depicted in **Figure** 3, are executed: (i) the GSMN bounds are set according to feedforward dependencies on compound tissue concentration; (ii) the objective function is optimized by FBA and associated example flux distribution is stored; (iii) the objective function value and/or solution fluxes are used to set the metabolism rate in the PBPK model; and (iv) compound concentration is updated by integration of the PBPK model ODEs within simulation time‐step. We note that nonunique FBA solutions may pose the challenge of applying this general approach in specific cases. If the GSMN model is not sufficiently constrained, the LP solver will be free to “flip” between alternative pathways corresponding to the same maximal value of objective function. If the PBPK metabolism rate is set to specific pathway fluxes, the arbitrary choices of the FBA solver between equivalent solutions may lead to discontinuity of ODE simulation between consecutive time‐steps. Thus, we would recommend the full protocol shown in **Figure** 3 to be used only in the case of tissue‐specific models being sufficiently constrained to produce a unique solution, at least for fluxes coupling GSMN and PBPK models. Otherwise, useful multiscale simulation can be constructed using exclusively feedforward or feedback coupling. For example, if the compound simulated by PBPK model is an enzyme inhibitor, and not metabolized by tissue represented in GSMN, only feedforward coupling (step 1) is needed to simulate the effect of a drug on metabolic capabilities. These capabilities may be evaluated as separate objective functions for which the LP solver would return unique maximal values. In another scenario, in which the drug is metabolized but does not inhibit any intracellular enzymes, the GSMN may be used to calculate the maximal drug metabolism rate as a function of genetic perturbations. In this case, the feedforward step is not needed and a unique objective function value can be used to set the metabolism rate in the PBPK model.

**Table 1:** Summary of multi‐scale models coupling GSMNs and ODE‐based models

Description	GSMN	ODE model	Objectives	Ref.
Pioneering approach of PBPK and GSMN integration	HepatoNet1 – liver model	Generic PBPK model created using parameter values available in the literature	Allopurinol effects on uric acid metabolism. Ammonia detoxification. Paracetamol toxicity.	45
Blood glucose regulation	Recon1‐derived adipocyte, hepatocyte, and myocyte GSMNs	PBPK model tailored for insulin‐glucagon‐glucose metabolism	Drug‐induced and genes knockout effects on whole body glucose levels	49
Levodopa and amino acid metabolism	Enterocyte specific GSMN (sIEC)	ACAT model (whole body PBPK model with advanced parametrization of gastrointestinal compartments)	Relationship between levodopa and amino acids metabolism and kinetics	50
MUFINS	Recon2	Glucose and lactate metabolic model and detailed CYP3A4 signaling model	Multiscale integration of GSMN, ODE‐based metabolic, and gene regulatory models	43

ACAT, advanced compartmental absorption and transit; CYP, cytochrome P450; GSMN, genome scale metabolic network; MUFINS, multiformalism interaction network simulator; ODE, ordinary differential equation; PBPK, physiologically based pharmacokinetic; sIEC, small intestine epithelial cell; WB‐ACAT‐sIEC, whole body advanced compartmental absorption and transit model (PBPK) combined with enterocyte specific GSMN model.

Gene regulation happens in timescales of hours and quasi‐steady‐state assumption has been extensively used in the systems biology field to couple dynamic models of gene regulation to GSMNs. The original, regulatory FBA method coupled Boolean GRN to FBA.^48^ Later, integrated FBA and integrated dynamic FBA methods extended this methodology to dynamic ODE models.^49^, ^50^ Given that timescales of gene regulation and drug absorption, distribution, and clearance are similar to those of gene regulation, the integration of gene regulation and PBPK should be performed within a dynamic modeling framework. In fact, simple gene induction models are already available in PBPK, opening the avenue for incorporation of more sophisticated networks from the systems biology field. Thus, in the quasi‐steady‐state multiscale model integrating PBPK, gene regulation, and whole‐cell metabolism, steady state should be assumed for GSMN, whereas both gene regulation and PBPK should be represented by dynamic model.

### Existing multiscale models integrating PBPK, GSMN, and gene regulation of metabolic enzymes

**Table** 1 ^43–46^ presents multiscale models that integrate PBPK and GSMN or GSMN and GRNs in human tissues. The example introduced below represents the first prototype of a PBPK model being integrated with both whole‐cell tissue‐specific metabolic model and ODE system describing gene regulation of drug metabolism enzyme.

Krauss *et al*.^44^ published the very first multiscale model integrating GSMN and PBPK to study the PKs of allopurinol, ammonia, and paracetamol in the context of a tissue‐specific model of liver metabolism (HepatoNet1)_ENREF_45. A method, similar to the Krauss *et al*.^44^ approach, was further incorporated in the subsequent two studies that used clinical data to parameterize healthy and diseased populations to fully use the multiscale modeling method.^45^, ^46^ This created multiscale whole body metabolic models to improve current therapeutic approaches for Parkinson disease treatment and suggested novel drug targets for treating type I diabetes.^46^ Additionally, a recently published multiscale modeling software package named MUFINS (multi‐formalism interaction network simulator) demonstrated several multiscale models as part of the software usability examples.^43^ The particular use case of the MUFINS software described a multiscale model consisting of GSMN model Recon2 coupled to an ODE‐based model of glucose and lactate metabolism and gene regulatory models. Although this model did not include PBPK *per se*, it did include physiological level variables describing blood concentrations, GSMN, and notably gene regulation of drug metabolism enzyme. The CBM and multiscale approaches discussed in this tutorial, together with their applicability under different circumstances, are summarized in **Table** 2. The used case below demonstrates simulation of a multiscale model integrating GSMN, gene regulation, and PBPK.

**Table 2:** Summary of CBM and multiscale methods discussed in this tutorial

Method	Questions addressed by the simulation	Considerations
Linear programming optimization	• Is particular tissue capable of metabolic function of interest? • What is maximal possible metabolic capability*?	The linear programming solver returns maximal value of objective function, but this value may correspond to many alternative intracellular flux distributions.
Reaction essentiality scan	• Which metabolic enzymes are possible targets for inhibition of metabolic function? • Polymorphism in which genes has an effect on metabolic function?	A “virtual screen” for potential drug targets, metabolic enzymes involved in adverse outcome pathways, and gene‐drug interactions.
Null space analysis	• Which pathways/enzymes/genes can potentially participate in metabolic function?	This method is based on enumeration of all possible pathways, which leads to combinatorial explosion in the case of large models. It is not practical for GSMNs.
Markov Chain Monte Carlo	• What is distribution of flux through particular intracellular reaction? • Which reactions are correlated?	Does not assume objective function. Computationally expensive as large samples are needed. The best way to explore intracellular flux distribution and its changes upon perturbation.
Feedback integration of CBM and PBPK	• Which metabolic enzymes have an effect on PK? • Polymorphism in which genes has an effect on PK?	If there is no feed forward integration, the linear programming needs to be evaluated only once and maximal metabolic capability can be used as parameter of PBPK model.
Feed forward integration of CBM and PBPK	• How does maximal metabolic capability change along PK time profile?	
Full integration of CBM and PBPK	• All questions answered by feedback and feed forward integration.	If the intracellular fluxes rather than objective function values are fed back to ODE system, numerical problems may be caused by LP solver arbitrarily switching between alternative solutions corresponding to the same objective function value. Full integration requires well constrained GSMN.
Integration of PBPK, CBM, and GRNs.	• All questions answered by feedback and feed forward integration. • How does the drug targeting transcription factor change intracellular metabolism and PK? • How does endogenous ligand binding transcription change intracellular metabolism and PK?	Well constrained GSMNs are required. Incorporation of gene regulation may help to constrain solution space.

CBM, constraint‐based modeling; GRN, gene regulatory network; GSMN, genome scale metabolic network; LP, linear programming; ODE, ordinary differential equation; PBPK, physiologically based pharmacokinetic; PK, pharmacokinetic.

^a^Metabolic capability is represented by objective function defined in Eq. (2).

## CASE STUDY

**Figure 4:**
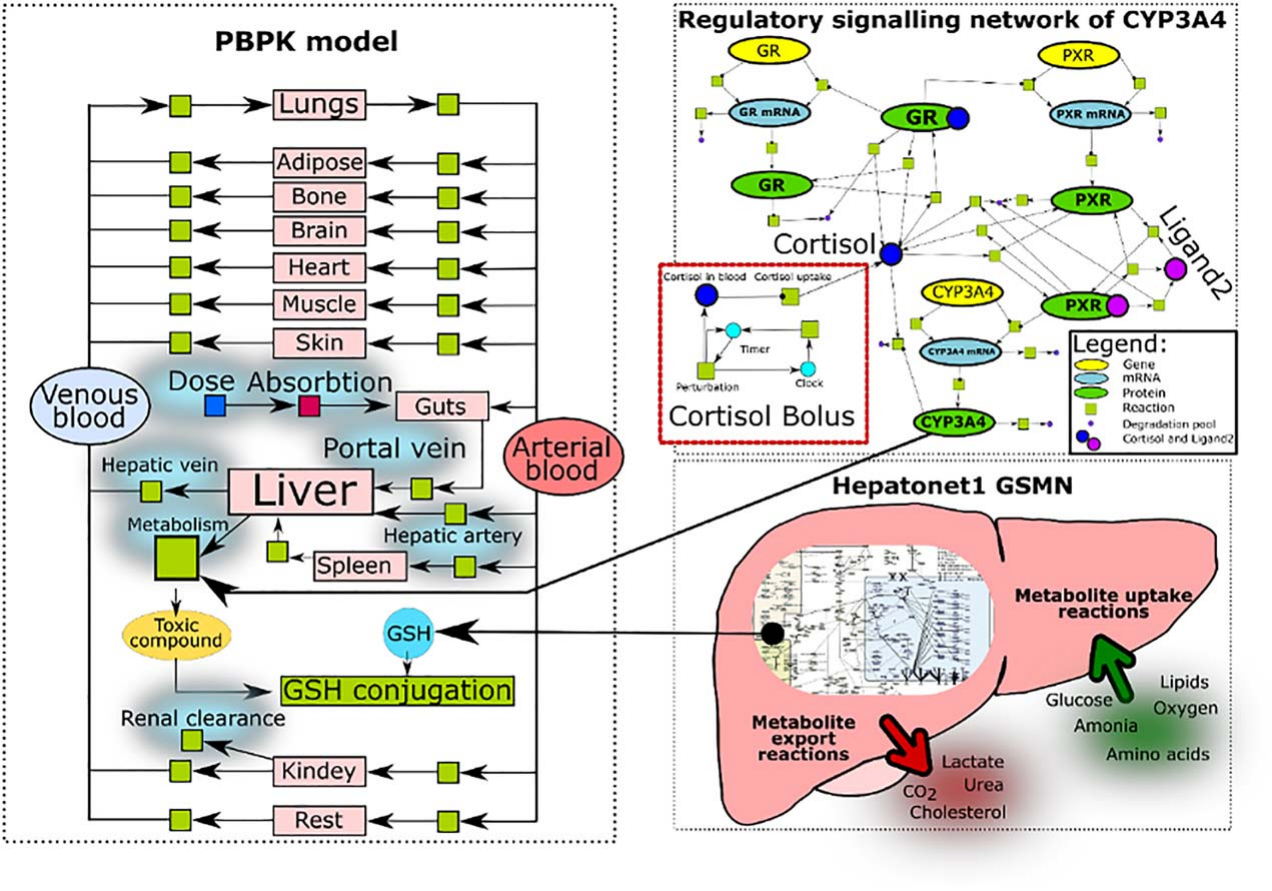
Multiscale model composed of physiologically based pharmacokinetic (PBPK), genome scale metabolic network (GSMN), and gene regulatory network. Figure schematically represents the multiscale model, created by combining the PBPK model with the gene regulatory network of cytochrome P450 (CYP)3A4 and human liver genome scale metabolic model HepatoNet1. The CYP3A4 model is further connected to the cortisol bolus module, which allows it to simulate chronic exposure to the stress. The regulatory network controls the total amount of CYP3A4 in the liver and defines the rate of drug being converted into the toxic metabolite. In addition, the total glutathione (GSH) availability is modeled by the HepatoNet 1 liver model. The total amount of GSH in the liver is proportionally scaled to the metabolic flux rate of GSH production at a steady‐state condition. Additionally, we have included the competitive inhibition of formyltetrahydrofolate dehydrogenase reaction dependent on the concentration of the metabolized example drug in the liver. The perturbations in the GSMN model, such as gene knockout or modulation of reactions fluxes (change of rate in the uptake reactions) or concentration‐dependent enzyme inhibition, allows the assessment of the impact on the metabolism of the toxic compound in the liver compartment of the PBPK model. The perturbations of the multiscale model evaluates the impact to the detoxification of the drug in response to the various physiological factors, such as stress (concentration of the cortisol in the blood), enzyme activity related to the genetic polymorphism, and molecular phenotype of the individual. GR, glucocorticoid receptor; PXR, pregnane X receptor.

We demonstrated the multiscale model consisting of three different modules: PBPK, GSMN, and gene regulation. We show the potential of this model to analyze the PK properties of the drug and its toxic metabolite in the context of various perturbations, such as exposure to chronic stress and patient‐specific liver metabolism (**Figure** 4). The multiscale model used in our example was built using MUFINS software and simulated using the qsspn simulator (available as part of the MUFINS package).^43^ The model and software required for its execution are available in the **Supplementary Material**.

We have used the human PBPK model with an example drug, as described in the previous tutorial of Jones & Rowland‐Yeo.^1^ We have extended this model by assuming that the drug under investigation is metabolized by cytochrome P450 (CYP)3A4 enzyme to a toxic metabolite. Although this system is motivated by a well‐studied example of paracetamol metabolism and N‐acetyl‐p‐benzochinonimin, we stress that we use example parameters within physiological ranges and do not attempt to model the particular compound. The model is meant to be an example of the simulation approach. The PBPK model was connected with HepatoNet1 GSMN.^51^ The nuclear receptor model controlling CYP3A4 was incorporated from a previously published study.^26^ Overall, we demonstrate how to connect three different models into one multiscale system capable of calculating drug metabolism in the context of whole human body, molecular liver metabolism, and nuclear receptor control (**Figure** 4).

### Example application 1: Discovery of metabolic reactions affecting dynamics of toxic metabolite

We have further constrained HepatoNet1 to increase quantitative accuracy of predictions. We have used a high throughput metabolomics dataset, published after original publication of HepatoNet1, in which consumption and release fluxes for over 200 metabolites were measured in all cancer cell lines in the NCI‐60 collection.^52^ Cancer cell lines divide much faster than cells of any human tissue and it is, therefore, reasonable to assume that no human tissue would have uptake and release fluxes with a faster rate than the maximum rate observed in cancer cell lines. We have, therefore, set upper and lower bounds of consumption and release fluxes according to the maximum consumption and release fluxes observed in the NCI‐60 cell lines. Moreover, we limited a set of nutrient consumption and release reactions to the physiological import and physiological export set defined for liver tissue in HepatoNet1 publication. Details of GSMN model parameterization are given in **Supplementary Text**.

The FBA allows the simulation of various liver functions, such as glutathione (GSH) production or conversion of ammonia to urea. In our example, the HepatoNet1 liver model was coupled to the PBPK model via GSH production flux, due to the GSH playing a crucial part in the detoxification of drugs activated by the phase I metabolism. We set GSH production as an objective function and, therefore, used unique maximal value calculated by LP. This value has been translated into scaling factor using the lookup table presented in **Supplementary Table S3**. In this particular case, the scaling factor was proportional to maximal GSH availability. The GSH conjugation rate in the PBPK model was then scaled using the following formula:

$$GSH\;conjugation\;rate=SF({GSH}_{\text{max}})*CLmet*\left(\frac{Atox}{Vli}\right)*fup$$

where, SF(GSH_max_) is a scaling factor proportional to the objective function value GSHmax calculated by LP of the GSMN model.

At this stage, the model involved only a feedback integration: result of FBA was used to scale parameters in the PBPK model. Because there was no change to the GSMN reaction flux bounds during dynamic simulation, only one evaluation of objective function was needed. Therefore, we could apply a reaction essentiality scan to identify reactions that affect GSH availability. In this *in silico* screening simulation, each reaction in turn was inactivated by setting flux bounds to (0, 0) and objective function was re‐evaluated. Reactions for which the objective function value was different from the value in the unperturbed model are shown in **Table** 3. As can be seen, the metabolic network is robust: only 41 reactions influence maximal GSH production. Each reaction listed in **Table** 1 ^43–46^ constitutes a hypothesis that certain intracellular enzyme affects GSH availability if subjected to inhibition by a drug or xenobiotic or if its activity is affected by genetic polymorphism. These reactions are potential targets for safety or DDIs. Their influence on the PBPK results can be further examined by creating a feedforward link between PBPK and GSMN.

**Table 3:** Reaction essentiality analysis results

Reaction	Flux rate	Reaction definition
*EX_Valine*	0	Valine import
*EX_Tyrosine*	0	Tyrosine import
*EX_Tryptophan*	0	Tryptophan import
*EX_Threonine*	0	Threonine import
*EX_Serine*	0	Serine import
*EX_Phenylalanine*	0	Phenylalanine import
*EX_Methionine*	0	Methionine import
*EX_Lysine*	0	Lysine import
*EX_L‐Lactate*	0	L‐Lactate export
*EX_Leucine*	0	Leucine import
*EX_Isoleucine*	0	Isoleucine import
*EX_Glutamine*	0	Glutamine import
*EX_Glucose*	0	Glucose import
*r0230*	0.011	5,10‐Methylene‐THF(c) + Glycine(c) + H2O(c) <=> THF(c) + Serine(c)
*r0293*	0.011	5,10‐Methylene‐THF(c) + NADP+(c) <=> 5,10‐Methenyl‐THF(c) + NADPH(c)
*r0663*	0.809	3‐Phosphoserine(c) + AKG(c) <– 3‐Phosphonooxypyruvate(c) + Glutamate(c)
*r0338*	0.809	3PG(c) + NAD+(c) –> 3‐Phosphonooxypyruvate(c) + NADH(c)
*r0159*	0.809	3‐Phosphoserine(c) + H2O(c) –> Serine(c) + Pi(c)
*r0227*	0.845	10‐Formyl‐THF(c) + H2O(c) + NADP+(c) –> THF(c) + CO2(c) + NADPH(c)
*r0371*	0.848	5,10‐Methenyl‐THF(c) + H2O(c) <=> 10‐Formyl‐THF(c)
*r0911*	0.867	Proline(c) + Glutamate(m) <=> Proline(m) + Glutamate(c)
*r1426*	0.868	Proline(m) + H+(PG)(c) –> Proline(c) + H+(PG)(m)
*r2539*	0.986	2‐Aminoadipate_6‐semialdehyde(c) + L‐2‐Aminoadipate(m) <=> 2‐Aminoadipate_6‐semialdehyde(m) + L‐2‐Aminoadipate(c)
*r0594*	0.986	2‐Aminoadipate_6‐semialdehyde(c) + NAD+(c) + H2O(c) <=> NADH(c) + L‐2‐Aminoadipate(c)
*r0450*	0.986	AKG(m) + L‐2‐Aminoadipate(m) <=> 2‐Oxoadipate(m) + Glutamate(m)
*r0180*	0.986	H2O(m) + Saccharopine(m) + NADP+(m) <=> AKG(m) + NADPH(m) + Lysine(m)
*r0658*	0.992	ATP(m) + 3‐Methylcrotonyl‐CoA(m) + HCO3‐(m) <=> ADP(m) + Pi(m) + 3‐Methylglutaconyl‐CoA(m)
*r0655*	0.992	Isovaleryl‐CoA(m) + Ubiquinone(m) <=> 3‐Methylcrotonyl‐CoA(m) + Ubiquinol(m)
*r0490*	0.992	HMG‐CoA(m) <=> 3‐Methylglutaconyl‐CoA(m) + H2O(m)
*r0262*	0.992	Leucine(m) + AKG(m) <=> Glutamate(m) + 4‐Methyl‐2‐oxopentanoate(m)
*r0779*	0.994	3‐Hydroxyisobutyryl‐CoA(m) + H2O(m) –> 3‐Hydroxyisobutyrate(m) + CoA(m)
*r0669*	0.994	Methacrylyl‐CoA(m) + H2O(m) –> 3‐Hydroxyisobutyryl‐CoA(m)
*r0560*	0.994	Isobutyryl‐CoA(m) + Ubiquinone(m) –> Methacrylyl‐CoA(m) + Ubiquinol(m)
*r0482*	0.994	3‐Hydroxyisobutyrate(m) + NAD+(m) –> 2‐Methyl‐3‐oxopropanoate(m) + NADH(m)
*r0263*	0.994	Valine(m) + AKG(m) –> Glutamate(m) + 3‐Methyl‐2‐oxobutyrate(m)
*r0240*	0.998	Threonine(c) <=> 2‐Oxobutyrate(c) + NH3(c)
*r0588*	0.999	(S)‐3‐Hydroxybutyryl‐CoA(m) <=> Crotonyl‐CoA(m) + H2O(m)
*r0541*	0.999	Glutaryl‐CoA(m) + Ubiquinone(m) <=> Crotonyl‐CoA(m) + CO2(m) + Ubiquinol(m)
*r0460*	0.999	(S)‐3‐Hydroxybutyryl‐CoA(m) + NAD+(m) <=> NADH(m) + Acetoacetyl‐CoA(m)

To demonstrate multiscale simulation involving both feedback and feedforward steps, we have assumed that toxic metabolite in the PBPK model inhibits one of the reactions limiting GSH availability. We established a further link between PBPK and GSMN modules by creating the competitive inhibition link of formyltetrahydrofolate dehydrogenase (FTHLDH) reaction (HepatoNet1 reaction name is r0027) using the formula below, which is further detailed by Krauss *et al*.^44^, ^46^

$$Reaction\;activity=\frac{1}{1+\frac{I\left(t\right)}{2*K_{i}}}$$

To calculate r0027 activity, we assumed the substrate concentration to be equal to the Km value and implemented Ki of 50 mM. The calculated reaction activity state was set to constrain r0227 reaction by implementing proportional reduction in the upper bound reaction flux rate to the corresponding reaction activity level (for example, the reaction activity status of 50% constrained the flux values from 0.0–1.0 to 0.0–0.5).

**Figure 5:**
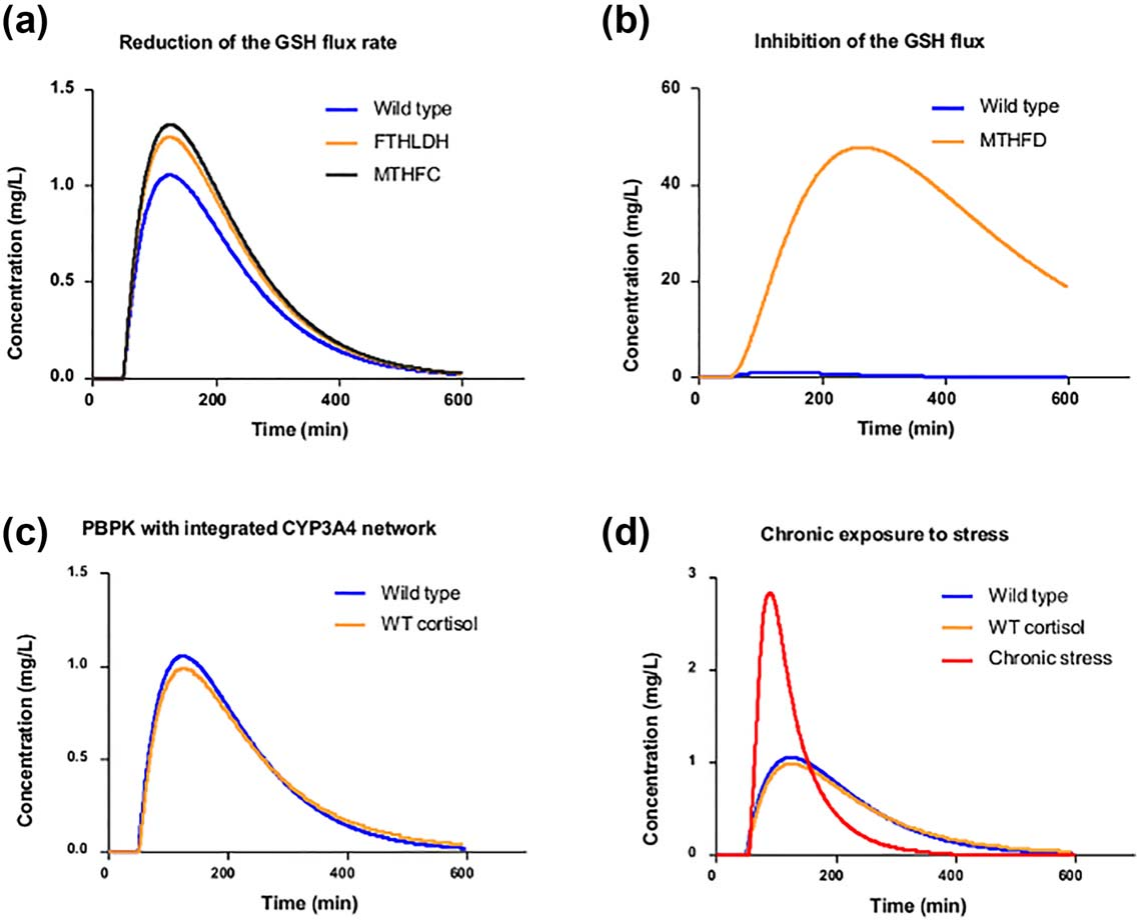
Metabolic analysis of the multiscale physiologically based pharmacokinetic (PBPK)/cytochrome P450 (CYP)3A4/liver model. The concentration of the toxic metabolite is calculated using the PBPK model and expressed as concentration (mg/L) within the liver compartment. (**a**) The unperturbed dynamic simulation of the PBPK model (blue line) calculates the dynamic rate of the toxic metabolite, which is subjected to the production, and glutathione (GSH) conjugation rates. The coupled liver genome scale metabolic network (GSMN) model calculates the GSH production rate at steady‐state conditions and perturbations of the GSH production rate impact the total GSH availability for the conjugation reaction. Inhibition of formyltetrahydrofolate dehydrogenase (FTHLDH) and MTHFC reactions reduces the GSH flux rate and, as a result, leads to the increased peak of the concentration of the toxic metabolite. (**b**) The effect of inactivating reaction identified in reaction essentiality scans of the GSMN. (**c**) The connected gene regulatory network under normal homeostatic conditions does not affect the rate of drug metabolism. (**d**) Simulation of the chronic stress conditions increase and sustains higher plasma cortisol concentrations, which leads to higher CYP3A4 expression rate. As a result, the increased CYP3A4 liver concentration causes significantly higher phase I metabolism rates and production of the toxic metabolite. MTHFC, methenyltetrahydrofolate cyclohydrolase; MTHFD, methylenetetrahydrofolate dehydrogenase.

Simulation results of the multiscale model are shown in **Figure** 5. **Figure** 5 **a** shows production of toxic metabolite under the unperturbed conditions (wild type). The inhibition of FTHLDH and methenyltetrahydrofolate cyclohydrolase (MTHFC) increases the concentration of the toxic metabolite via reduction of the GSH flux rate. Additionally, the inactivation of reaction methylenetetrahydrofolate dehydrogenase (MTHFD) completely blocks the production of the GSH in the liver model and, as a result, severally impacts the detoxification of the toxic metabolite (**Figure** 5 **b**). These results should prompt further examination of genetic polymorphism in FTHLDH, MTHFC, and MTHFD to consider whether there are genetic backgrounds particularly vulnerable to administration of the drug. In addition, compounds inhibiting FTHLDH, MTHFC, or MTHFD are likely to interact with the drug by increasing the concentration of its toxic metabolite.

### Example application 2: Integration of human hepatocyte GSMN, gene regulation of CYP3A4, and basic PBPK

In the second example, we further enhance the PBPK‐GSMN model by adding the GRN, which controls liver CYP3A4 concentration in response to the cortisol‐induced signaling. Previously established dynamic ODE‐based CYP3A4 regulatory network was connected with the PBPK model to regulate the rate of the phase I metabolism by controlling CYP3A4 levels in response to cortisol signaling.^26^ Therefore, the addition of the CYP3A4 regulatory network allows the investigation of the impact of environmental perturbation, such as stress to the metabolism and assesses the possible toxicity to the susceptible population. The rate of drug metabolism within the PBPK model was coupled to the total CYP3A4 amount in the liver (705 nM), the percentage increase or decrease in CYP3A4 concentration directly correlated with changes in the rate of drug metabolism (i.e., 10% increase in CYP3A4 amount from 705 nm to 775 nM lead to 10% increase in rate of drug metabolism). The connected CYP3A4 regulatory network does not impact metabolism of the drug under the normal homeostatic conditions (**Figure** 5 **c**). In contrast, the perturbation of CYP3A4 homeostatic regulation by simulating chronic stress led to the drastic increase of the toxic metabolite concentration (**Figure** 5 **d**). Chronic stress conditions increases and sustains higher blood cortisol concentration, which impacts nuclear receptor signaling. As a result, the increased cortisol signaling pathway leads to the perturbation of homeostatic CYP3A4 concentration (increase) and in turn elicits an impact to the metabolism of the drug.

## FUTURE PERSPECTIVES

In this tutorial, we have reviewed pioneering literature describing multiscale models integrating PBPK and two classes of models from the systems biology field: GSMNs and quantitative dynamic models of GRNs. We have constructed example models and made them available in free software. We note that although most applications of quantitative systems pharmacology focus on pharmacodynamics, this tutorial shows how legacy of systems biology can be used to increase mechanistic scope of literature‐based, bottom‐up PK modeling.

We believe that the area of multiscale modeling presented here will expand in the future due to the following motivation. First, the advent of next generation sequencing brings full genome sequencing of individual patients closer to clinical reality. This will motivate identification of intracellular metabolism enzymes that indirectly affect PK to fully use patient's genetic information in personalized medicine context. Our example model shows identification of reactions that affect toxic metabolite dynamics indirectly through limitation of GSH production in liver cells. In population context, meta‐analysis of data collected in genomewide association studies can be used to determine distribution of genetic polymorphism in the population.^53^ To make full use of these data, mechanistic models need to include, in principle, variables for all genes in the genome. Literature and examples presented here show how the extension of PBPK by models of intracellular metabolism, mechanistically accounting for thousands of human genes, brings this closer to reality. Another important motivation comes from the growing area of quantitative systems toxicology. Contrary to pharmacology, in which the focus is on a single compound optimized for specific binding to a known target or a combination of a few such compounds, toxicology deals with potentially weak, less specific binding of possibly a large number of compounds to multiple targets. Again, to address this challenge, molecular networks operating in human tissues need to be mechanistically modeled. We show an example model addressing this challenge by both incorporation of whole‐cell metabolism and steady state as well as detailed quantitative model of the GRN playing a key role in the response to xenobiotics. Last but not least, biosimulation is gaining importance in the general area of health care, in which mechanistic models can be used in the future to support decisions about diet and lifestyle. Although this is probably the most challenging task for mechanistic modeling, we expect that PBPK formalism and parameters will find applications for modeling of diet components and substances made by the body, such as cortisol. Our example shows a simulation of how compound PK is related to cortisol burst, which may be induced by stress or other patient's conditions.

The success of quantitative, mechanistic modeling depends on availability of quantitative experimental data. Here, we show how advances in ∼omics technologies can be already used to quantitatively parameterize mechanistic models of unprecedented scale. In our example, we use the HepatoNet1 model that has been made liver‐specific by analysis of gene expression data. We have further constrained this model by providing biologically realistic, quantitative, consumption, and release flux bounds for over 200 metabolites, based on *in vitro* metabolomics dataset. We hope that development of genome‐scale mechanistic models relevant to pharmacology, toxicology, and health will motivate quantitative biology research, leading to increased quantitative accuracy of the models. We note that the CBM approach is particularly suitable in this context as it allows to gradually increase quantitative detail as new data become available.

In conclusion, we are convinced that multiscale models integrating PBPK and GSMNs operating in human tissues will gain importance in the near future. We hope that our tutorial provides a useful introduction to this exciting field.

## Supporting information

Supporting Information

Supporting Information
