# Supplementary material for: Integration of Genome Scale Metabolic Networks and Gene Regulation of Metabolic Enzymes With Physiologically Based Pharmacokinetics
Source: CPT Pharmacometrics Syst Pharmacol. 2017 Sep 8;6(11):732–46. doi: 10.1002/psp4.12230 (PMC5702902; doi:10.1002/psp4.12230)
Supplement: Supplementary file 1 — Supporting Information [file PSP4-6-732-s001.pdf]

Model construction

HepatoNet1 [1], a GSMN representing human hepatocyte whole-cell metabolism was sourced from BioModels (MODEL1009150000, SBML L2 V4). Adjustments were made to HepatoNet1 to better represent human hepatocyte monosaccharide metabolism (Table 1). Namely, HepatoNet1 reaction IDs: r0206 (KHK, EC 2.7.1.3), r0252 (TRIOK, EC 2.7.1.28), and r0554 (ALDOB, EC 4.1.2.13) were adjusted as shown in Table 1 according to the KEGG fructose and mannose pathway hsa00051, and protein knowledgebase UniProtKB [2-4]. In addition, a metabolite sorbitol, and metabolic reactions sorbitol dehydrogenase (SORD, EC 1.1.1.14) and aldo-keto reductase family 1, member B1 (AKR1B1, EC 1.1.1.21) were added to represent the polyol pathway ,[5] and hepatic fructose sodium co-transporter reaction (SLC5A9, TCDB 2.A.21.3.17); also found in the Recon 2 liver hepatocyte GSMN [6].

Table 1. Adjustments to HepatoNet1 GSMN.

Modified reactions

ID	*HepatoNet1 Reaction*	*Modified Reaction*
r0206	ATP(c) + Fructose(c) ← ADP(c) + Fructose-1P(c)	ATP(c) + Fructose(c) → ADP(c) + Fructose-1P(c)
r0252	ATP(c) + Glyceraldehyde(c) ↔ ADP(c) + GAP(c)	ATP(c) + Glyceraldehyde(c) → ADP(c) + GAP(c)
r0554	Fructose-1P(c) → DHAP(c) + Glyceraldehyde(c)	Fructose-1P(c) ↔ DHAP(c) + Glyceraldehyde(c)

Added Reactions

ID	*Reaction*
r9000	Glucose(c) + NADPH(c) → NADP(c) + Sorbitol(c)
r9001	NAD(c) + Sorbitol(c) → Fructose(c) + NADH(c)
r9002	Fructose(s) + Na^+(s) → Fructose(c) + Na^+(c)

Constrained fluxes[1]

ID	*HepatoNet1 Reaction*	*Lower Bound*	*Upper Bound*
r0356	Fructose(c) + ATP(c) → Fructose-6P(c) + ADP(c)	0.0	0.005
r0357	Fructose(c) + ITP(c) → Fructose-6P(c) + IDP(c)	0.0	0.005
r0358	Fructose(c) + dATP(c) → Fructose-6P(c) + dADP(c)	0.0	0.005
r9000	Glucose(c) + NADPH(c) → NADP(c) + Sorbitol(c)	0.0	0.000714

[1]The lower and upper bounds were set as flux ratios relative to glucose phosphorylation reactions r0353-5 set as 1. These flux constraints were based on enzyme K_m's described in Stocchi et al., 1982, Tanimoto et al., 1991, Sato et al., 1995, and Brown et al., 2005 as sourced from enzyme database, BRENDA. Abbreviations: cytosol (c), sinusoidal space (s), glyceraldehyde 3-phosphate (GAP), and dihydroxyacetone phosphate (DHAP).

HepatoNet1 external exchange set, physiological import and physiological export set (PIPES), was utilised here. The PIPES was parameterised to represent an *in vitro* system with mass spectrometry metabolite flux data observed in NCI60 cancer cell lines [7].The metabolites that were identified in both the PIPES and CORE dataset were constrained to the maximal consumption and release flux rates set as the lower and upper flux bounds, respectively. All flux rates were converted to mmol/gram of cell dry weight/hour, thus all output concentrations are mmol/gram of cell dry weight.

Table 2. The PIPES external metabolite exchange set constrained to NCI60 CORE.

Reaction ID	Reaction	Lower Bound	Upper Bound	Description
EX_H2O	HC00011_s = HC00011_s_xt	-1	1	H2O exchange
EX_O2	HC00017_s = HC00017_s_xt	-1	0	O2 import
EX_Pi	HC00019_s = HC00019_s_xt	-1	1	Pi exchange
EX_CO2	HC00021_s = HC00021_s_xt	0	1	CO2 export
EX_NH3	HC00024_s = HC00024_s_xt	-1	0	NH3 import
EX_Alanine	HC00048_s = HC00048_s_xt	-0.0055	0.130813	Alanine exchange

EX_Arginine	HC00065_s = HC00065_s_xt	-0.070285	0.005786	Arginine exchange
EX_Asparagine	HC00148_s = HC00148_s_xt	-0.037002	0.005679	Asparagine exchange
EX_Aspartate	HC00055_s = HC00055_s_xt	-0.017289	0.016442	Aspartate exchange
EX_Glutamate	HC00034_s = HC00034_s_xt	-0.007645	0.193094	Glutamate exchange
EX_Glutamine	HC00067_s = HC00067_s_xt	-0.608543	-0.027744	Glutamine import
EX_Glycine	HC00045_s = HC00045_s_xt	-0.011301	0.023408	Glycine exchange
EX_Isoleucine	HC00334_s = HC00334_s_xt	-0.043197	-0.002126	Isoleucine import
EX_Leucine	HC00121_s = HC00121_s_xt	-0.05223	-0.003955	Leucine import
EX_Lysine	HC00053_s = HC00053_s_xt	-0.038469	-0.005086	Lysine import
EX_Methionine	HC00075_s = HC00075_s_xt	-0.014031	-0.001399	Methionine import
EX_Phenylalanine	HC00081_s = HC00081_s_xt	-0.01461	-0.001669	Phenylalanine import
EX_Proline	HC00145_s = HC00145_s_xt	-0.002389	0.011564	Proline exchange
EX_Serine	HC00068_s = HC00068_s_xt	-0.067881	-0.004097	Serine import
EX_Threonine	HC00179_s = HC00179_s_xt	-0.024476	-0.002757	Threonine import
EX_Tryptophan	HC00080_s = HC00080_s_xt	-0.006982	-0.000055	Tryptophan import
EX_Tyrosine	HC00085_s = HC00085_s_xt	-0.021401	-0.002104	Tyrosine import
EX_Valine	HC00174_s = HC00174_s_xt	-0.03152	-0.003558	Valine import
EX_Choline	HC00112_c = HC00112_c_xt	-0.004596	0.000521	Choline exchange
EX_Folate	HC00396_s = HC00396_s_xt	-0.000046	0.000198	Folate exchange
EX_Nicotinamide	HC00149_s = HC00149_s_xt	-0.001838	0.000057	Nicotinamide exchange
EX_Pantothenate	HC00568_s = HC00568_s_xt	-0.000075	0.000005	Pantothenate exchange
EX_Urate	HC00310_s = HC00310_s_xt	-0.000306	0.000212	Urate exchange
EX_Glucose	HC00040_s = HC00040_s_xt	-1.723855	-0.077802	Glucose import
EX_L-Lactate	HC00177_s = HC00177_s_xt	0.064699	2.690282	L-Lactate export
EX_Cystine	HC00389_s = HC00389_s_xt	-1	1	Cystine exchange
EX_Histidine	HC00133_s = HC00133_s_xt	-1	1	Histidine exchange
EX_Fe2+	HC01846_s = HC01846_s_xt	-1	1	Fe2+ exchange
EX_Pyridoxine	HC00268_s = HC00268_s_xt	-1	0	Pyridoxine import
EX_Riboflavin	HC00232_s = HC00232_s_xt	-1	0	Riboflavin import
EX_Cholesterol	HC00178_b = HC00178_b_xt	0	1	Cholesterol export
EX_H2S	HC00250_s = HC00250_s_xt	0	1	H2S export
EX_Sulfate	HC00062_s = HC00062_s_xt	-1	1	Sulfate exchange
EX_Urea	HC00089_s = HC00089_s_xt	0	1	Urea export
EX_Cysteine	HC00099_s = HC00099_s_xt	-1	1	Cysteine exchange
EX_Ethanolamine	HC00180_c = HC00180_c_xt	-1	1	Ethanolamine exchange
EX_Arachidonate	HC00202_s = HC00202_s_xt	-1	1	Arachidonate exchange
EX_Palmitate	HC00226_s = HC00226_s_xt	-1	1	Palmitate exchange
EX_Oleate	HC00510_s = HC00510_s_xt	-1	1	Oleate exchange
EX_Stearate	HC00793_s = HC00793_s_xt	-1	1	Stearate exchange
EX_Linoleate	HC00796_s = HC00796_s_xt	-1	1	Linoleate exchange
EX_Palmitolate	HC01981_s = HC01981_s_xt	-1	1	Palmitolate exchange

Multiscale model integrating GSMN, PBPK and gene regulation of drug metabolism enzyme. We provide all files which are required to simulate multiscale model used in our case studies with our published MUFINS software (http://sysbio3.fhms.surrey.ac.uk/mufins/). The detailed information of how to use MUFINS software is provided in the supplementary information of the original publication (qsspn_tutorial.pdf), therefore will not be repeated in this text [8].

Model distribution

Table below summarises describes files accompanied in the supplementary material.

File	Purpose/definition
qsspn.exe	QSSPN: Simulation engine of MUFINS software compiled in Cygwin.
CLEAN.bat	Deletes files generated by the last simulation

COMPILE.bat	Converts spept file to the qsspn file.
HepatoNet1_EMM_14122016.sfba	GSMN model
PBPK_GSMN_NR.ctrl.txt	Simulation control file
PBPK_GSMN_NR.qsspn	Multi-scale model in qsspn format.
PBPK_GSMN_NR.spept	Multi-scale model
RUN.bat	Runs simulation
spept2node.py	Python script converting snoopy file to qsspn
*.dll	Library files required to run Cygwin version of qsspn solver.

To run simulation execute the following commands in command prompt of Windows operating systems: i) COMPILE.bat to convert *.spept file into *.qsspn file. ii) RUN.bat to run simulation iii) examine simulation trajectories in output.xls file. To examine or modify the model, learn MUFINS software from material associated with original publication. Briefly, you will need to install Snoopy Petri Net editor and learn structure of control file.

Model building

PBPK and gene regulatory ODE based models were assembled using snoopy graphical user interface software (PBPK_GSMN_NR.spept file), while the propensity functions and initial system parameters were entered to the control file (PBPK_GSMN_NR.ctrl.txt). The Snoopy file (*.spept) and control files (*.ctrl.txt) are two arguments read by the qsspn simulator. Once the ODE models were assembled, the GSMN model was added. Fully assembled model needs to be compiled to the qsspn format before simulation can take place.

Short summary of model building and simulation workflow:

1) Reconstruct ODE models using snoopy petri-net
2) Add the GSMN to the snoopy petri-net model
3) Construct the control file, add all variable and write propensity functions
4) Compile the spept file to qsspn by executing COMPILE.bat
5) Run simulations executing RUN.bat
6) Before next simulation clean output by running CLEAN.bat

Look-up tables

GSH flux rate	GSH scaling factor	GSH value within the reaction
1.0	100%	1
0.95	95%	0.95
0.9	90%	0.9

0.84	84%	0.84
0.8	80%	0.8
0.7	70%	0.7
0.6	60%	0.6
0.5	50%	0.5
0.4	40%	0.4
0.02	2%	0.02
0.01	1%	0.01
0.001	0.1%	0.001
0.0	0%	0

Table 3 GSH bioavailability look-up table

**Example drug amount (g)**	**Reaction activity**	**r0227 flux values**	
		**LB**	**UB**
0.0	100%	0.0	1.0
1.0	89.1%	0.0	0.9
2.0	80.3%	0.0	0.8
3.0	73.1%	0.0	0.7
5.0	61.9%	0.0	0.6
6.0	57.6%	0.0	0.5
12.0	40.4%	0.0	0.4
15.0	35.2%	0.0	0.35
20.0	28.9%	0.0	0.29
32.0	20.3%	0.0	0.2
100.0	7.5%	0.0	0.0

Table 4 r0227 inhibition by the example drug look-up table

References

1. Gille, C., et al., *HepatoNet1: a comprehensive metabolic reconstruction of the human hepatocyte for the analysis of liver physiology.* Molecular Systems Biology, 2010. 6(1): p. 411.

2. Asipu, A., et al., *Properties of normal and mutant recombinant human ketohexokinases and implications for the pathogenesis of essential fructosuria.* Diabetes, 2003. 52(9): p. 2426-2432.

3. Beutler, E. and E. Guinto, *Dihydroxyacetone metabolism by human erythrocytes: demonstration of triokinase activity and its characterization.* Blood, 1973. 41(4): p. 559-568.

4. Santamaria, R., et al., *Functional and molecular modelling studies of two hereditary fructose intolerance-causing mutations at arginine 303 in human liver aldolase.* Biochemical Journal, 2000. 350(3): p. 823-828.

5. El-Kabbani, O., C. Darmanin, and R.-T. Chung, *Sorbitol dehydrogenase: structure, function and ligand design.* Current medicinal chemistry, 2004. 11(4): p. 465-476.

6. Thiele, I., et al., *A community-driven global reconstruction of human metabolism.* Nature biotechnology, 2013. 31(5): p. 419-425.

7. Jain, M., et al., *Metabolite profiling identifies a key role for glycine in rapid cancer cell proliferation.* Science, 2012. 336(6084): p. 1040-1044.

8. Wu, H., et al., *MUFINS: Multi-Formalism Interaction Network Simulator.* npg Systems Biology and Applications, 2016. *2*: p. 16032.
